# Effect of Different Interferonα2 Preparations on IP10 and ET-1 Release from Human Lung Cells

**DOI:** 10.1371/journal.pone.0046779

**Published:** 2012-10-08

**Authors:** Rekha Badiger, Jane A. Mitchell, Hime Gashaw, Neil A. Galloway-Phillipps, Stefan Foser, Fernando Tatsch, Thomas Singer, Trevor T. Hansel, Tobias Manigold

**Affiliations:** 1 Cardiothoracic Pharmacology, National Heart and Lung Institute (NHLI), Imperial College, London, United Kingdom; 2 Hoffmann-La Roche, Basel, Switzerland; 3 Imperial Clinical Respiratory Research Unit (ICRRU), St. Mary's Hospital, London, United Kingdom; 4 Department of Internal Medicine, University Hospital, Basel, Switzerland; University of Leuven, Rega Institute, Belgium

## Abstract

**Background:**

Alfa-interferons (IFNα2a, IFNα2b, 40KDa-PEGIFNα2a and 12KDa-PEGIFNα2b) are effective treatments for chronic hepatitis C infection. However, their usage has been associated with a variety of adverse events, including interstitial pneumonitis and pulmonary arterial hypertension. Although rare, these adverse events can be severe and potentially life-threatening, emphasizing the need for simple biomarkers of IFN-induced lung toxicity.

**Methods:**

Human lung microvascular endothelial cells (HLMVEC), human pulmonary artery smooth muscle (HPASM) cells and A549 cells were grown under standard conditions and plated into 96- or 6-well plates. Cells were stimulated with various concentrations of different IFNs in hydrocortisone-free medium. After 24 and 48 hours, IP10 and ET-1 were measured by ELISA in conditioned medium. In a second set of experiments, cells were pre-treated with tumour necrosis factor-α (TNF-α) (10 ng/mL).

**Results:**

IFNα2a, IFNα2b, 40KDa-PEGIFNα2a and 12KDa-PEGIFNα2b, but not IFNλ, induced IP10 (CXCL10) release and increased IP10 gene induction in HLMVEC. In addition, all four IFNα preparations induced IP10 release from HPASM cells and A549 cells pre-treated with TNFα. In each of these cell types, 40KDa-PEGIFNα2a was significantly less active than the native forms of IFNα2a, IFNα2b or 12KDa-PEGIFNα2b. Similarly, IFNα2a, IFNα2b and 12KDa-PEGIFNα2b, but not 40KDa-PEGIFNα2a, induced endothelin (ET)-1 release from HPASM cells.

**Conclusions:**

Consistent with other interstitial pulmonary diseases, both IP10 and ET1 may serve as markers to monitor IFN-induced lung toxicity in patients. In addition, both markers may also serve to help characterize the risk associated with IFNα preparations to induce lung toxicity.

## Introduction

Interferons (IFNs) are potent cytokines involved in innate and adaptive immune responses [1], [2]. The immunomodulatory and antiviral properties of alfa interferons (IFNα) have been exploited therapeutically to treat a number of diseases, including chronic hepatitis C (CHC). In its native form, IFNα is relatively unstable and requires frequent parenteral administration. Pegylation of IFNα, where polyethylene glycol (PEG) molecules are bound to the native protein, has been shown to reduce *in vitro* activity but increase the stability and plasma half-life of IFNα [3], and have therefore largely replaced conventional IFNα in CHC treatment [4]. There are two forms of PEGIFNα used clinically; 40KDa-PEGIFNα2a (PEGASYS, Roche, Basel, Switzerland) and 12KDa-PEGIFNα2b (PegIntron, Merck, Whitehouse Station, NJ, USA), in which IFNα is conjugated to 40KDa and 12KDa PEG moieties, respectively. Importantly, the composition of therapeutic IFN preparations with different isomers may be crucial for efficacy [5]. Specifically, 40KDa-PEGIFNα2a consists of nine different monopegylated isomers that have a range of antiviral-specific activities that correlate with antiproliferative but not with global gene transcriptional activity in the melanoma cell line ME15 [5].

Administration of IFNα has been associated with a variety of adverse events, including pulmonary side effects [6]. These side effects, which include pneumonitis [6] and pulmonary arterial hypertension (PAH) [7], are rare but can be severe. Moreover, the most common clinical sign for IFNα-induced pneumonitis is cough [6], [8]. Both the severity and the low incidence of acute pneumonitis (0.02% [9]) and PAH, coupled with the lack of a specific symptom, indicate the need for biomarkers that allow monitoring of host inflammation in IFNα-treated patients. However, no biomarker is currently available to monitor the risks of IFN-induced lung toxicity.

The underlying mechanism leading to interstitial pneumonitis following IFNα is not clearly understood, but some recent studies in mice and humans have shed some light onto the pathophysiology of the condition. Recent studies have identified the role of CXCR3+ immune cells along with its cognate ligands, IFNγ-induced protein 10 (IP10; also termed CXCL10) and monokine induced by IFNγ (MIG; also termed CXCL9), in promoting migration of CXCR3+ cells to the lung and in lung inflammation [10]–[14]. Accordingly, different groups have found an association between IP10 [15]–[18] or endothelin 1 (ET-1) [19] and different interstitial lung diseases (ILDs).

One question that needs to be addressed is how type I IFNs initiate inflammatory processes in patients. Type I IFNs act via the ubiquitously expressed IFNα receptor (IFNAR) complex, which has two components, IFNAR1 and IFNAR2. Type II IFNγ binds to the IFNγ receptor complex and mediates innate immune responses to bacteria and viruses. Less is understood about type III IFNλ, but it is known to signal via IFNλ receptor 1 and interleukin-10 receptor 2 [20]. Activation of IFN receptors leads to the induction of IFN-selective genes, of which IP10 is a principle example. However, there is recent evidence from studies with mice and human endothelial cells that not only type II IFNs but also type I IFNs can induce IP10 [21]. Given the emerging importance of the association between IP10 and ILDs, an important question remains regarding whether type I IFNs could induce IP10 in human lung cells. However, IP10 may not be the only important factor contributing to ILDs.

A second mediator of interest for the development of ILDs is ET-1. We have previously shown that IFNγ induces ET-1 in tumour necrosis factor-α (TNFα)-primed human pulmonary artery smooth muscle cells [22]–[25] and smooth muscle cells from human systemic vessels [26]. ET-1 is a critical mediator and therapeutic target in PAH and is associated with remodelling and vasoconstriction [27]. Accordingly, associations were found between broncho-alveolar lavage fluid (BALF) and serum levels of ET-1 in several human lung diseases [27]–[29].

Thus, we hypothesized that type I IFNs lead to IP10 and possibly ET-1 release from human lung tissue. This could provide the basis for CXCR3+ immune cell migration to the lung and the promotion of lung inflammation. Consequently, IP10 and ET-1 could be useful biomarkers to indicate increased risk of IFNα-induced interstitial pneumonitis. Pneumonitis occurs when fluid shifts across leaky alveoli, implicating alveoli epithelium. Endothelial damage is also implicated in pneumonitis [30]. In pulmonary hypertension, endothelial cells and the underlying vascular smooth muscle cells are targets for inflammation and cellular dysfunction. We investigated the effect of therapeutic and non-therapeutic IFNα preparations on IP10 and ET-1 release by relevant human lung cells *in vitro*.

## Methods

### Cell culture and treatments

Human lung microvascular endothelial cells (HLMVEC) were grown and maintained in specific media according to the manufacturer's instructions (Lonza, Basel, Switzerland). Four days prior to treatments, hydrocortisone was withdrawn from culture conditions in order to study the release of inflammatory mediators. Human pulmonary artery specimens were obtained from healthy segments of lung from patients undergoing lung resection at the Royal Brompton Hospital, London, UK (Research Ethics Committee study number 02-081, sub-amendment 3). Full informed written consent was obtained from all participants.

Human pulmonary artery smooth muscle (HPASM) cells were grown in Dulbecco's modified Eagle's medium (DMEM, Sigma), supplemented with 15% heat- inactivated fetal calf serum (FCS), L-glutamine (2 mM), streptomycin (100 µg/mL), penicillin (100 U/mL) and 1% vol/vol 100× MEM non-essential amino acids (added according to manufacturer's instructions; GIBCO Life Technologies, Paisley, Renfrewshire, UK). Serum was withdrawn for 24 hours prior to treatments; treatments were then carried out in media with 10% FCS. A549 cells (ECACC, Salisbury, Wiltshire, UK) were grown, maintained and treated in medium containing 10% FCS. For HLMVEC and HPASM cells, cells less than passage 8 were used. Cells were plated and treated using either standard 96-well or 6-well plates. Human recombinant forms of IFNα (universal type I IFN), IFNβ, IFNγ and IFNλ were from R&D Systems (Abingdon, Oxfordshire, UK). Human IFNα2a, IFNα2b, 40KDa-PEGIFNα2a, 12KDa-PEGIFNα2b, and 40KDa-PEGIFNα2a isoforms K31, K134 and K122 [5] were provided by Hoffmann-La Roche (Basel, Switzerland). In experiments with TNFα co-treatment, TNFα was added 10 minutes prior to the IFNs at a dose of 10 ng/mL.

### Measurement of IP10 and ET-1

Conditioned media was collected at 24 and 48 hours for analysis. ET-1 and IP10 were measured using specific commercial ELISA kits from R&D systems.

### Measurement of IP10 and related genes

For gene expression experiments, cells were treated in 6-well plates, with each well representing a different treatment; cells were treated for 6 hours before RNA was extracted. Each well of the 6-well plate yielded sufficient RNA for PCR amplification, typically a quantity of 2–3 µg for HLMVEC and 5–10 µg for HPASM cells. RNA was extracted from the cells using a commercial RNA extraction kit according to the manufacturer's protocol (Quiagen, Crawley, West Sussex, UK). RNA quantification was performed using Nanodrop 2000c UV spectrophotometery (Thermo Scientific, Epsom, Surrey, UK), following which the samples were stored at −80°C prior to use in PCR experiments. Gene expression was measured using the Inflammatory Cytokines and Receptors PCR Array from Qiagen, which contains pre-dispensed gene-specific primer sets for 84 inflammation-related genes. Seven genes in each plate were related to PCR quality and DNA contamination controls and 5 genes in the array were housekeeping genes. Gene expression differences between IFN-treated and control samples were calculated using the equation 2*(−ΔΔCT).

### Measurement of cell viability in A549 and HLME cells

Cell viability was measured using the MTT assay. In the absence or presence of TNFα, IFNs (at the top concentration used after 48 hrs) had no additional effect on cell viability. This suggests that IFNs are not toxic *per se* to these cells.

## Results

### Effect of type I IFNα and IFNβ, type II IFNγ and type III IFNλ on IP10 and ET-1 release by human lung cells

**Figure 1:**
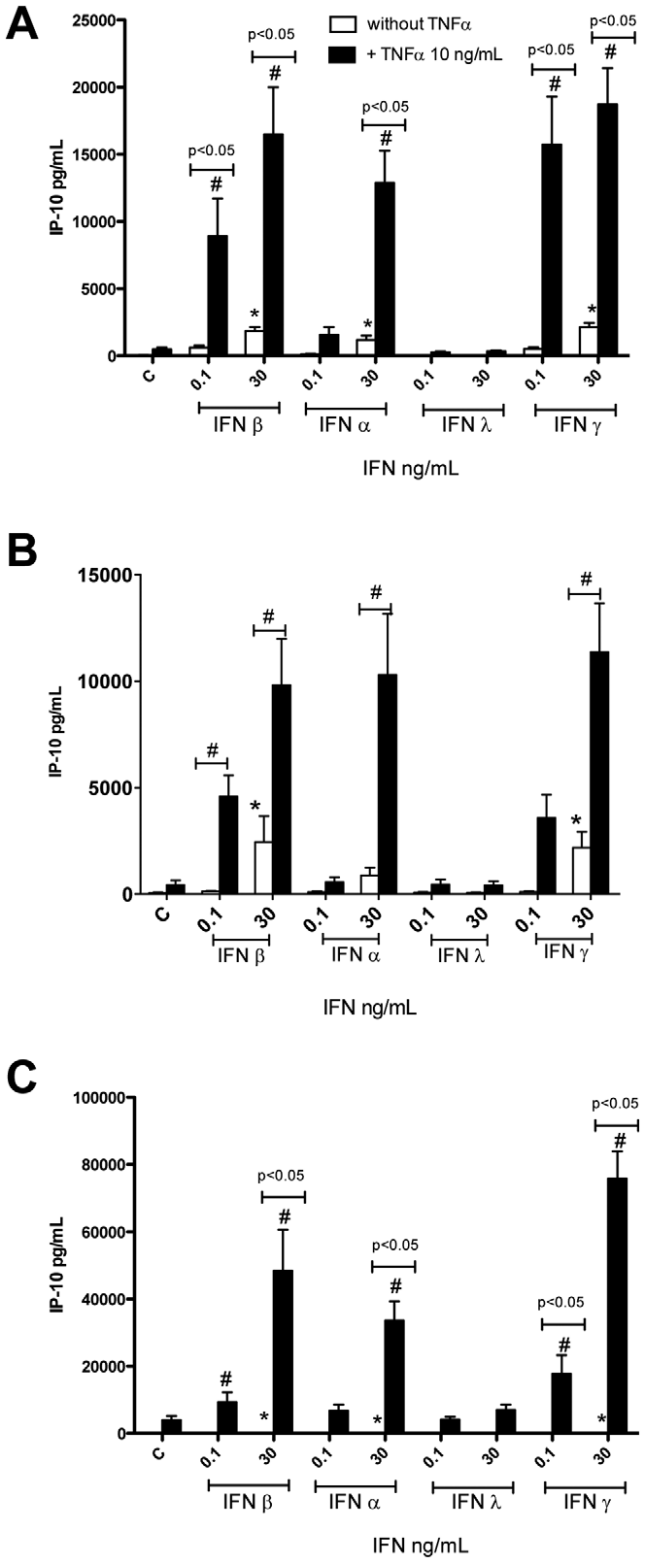
Effect of type I–III IFNs on IP10 release by human lung cells. In the presence of TNFα, type I and type II, but not type III, interferons (IFNs) induce the release of IP10 from endothelial cells (HLMVECs; A), human pulmonary artery cells (HPASMCs; B) and human type II pneumocytes (A549 cells; C). Cells were treated with IFNs for 24 hours. Data are the mean ± standard error of the mean for n = 6–8. Within-group analysis was performed using one-way ANOVA followed by a Dunnett's post-test, where * indicates p<0.05 compared to control. Between-group analysis, for the effect of TNFα was performed using two-way ANOVA followed by Bonferroni's post-test where # indicates p<0.05.

Under control culture conditions (without TNFα), IP10 release by all cell types studied was very low/undetectable (**Figure 1**). However, IFNα (universal type I IFN), IFNβ and IFNγ, but not IFNλ increased levels of IP10 from HLMVEC and HPASM cells. The release of IP10 was increased when cells were pre-treated with TNFα (**Figure 1**). IP10 release from A549 cells was very low after IFN stimulation alone, but was dramatically increased by TNFα (**Figure 1**).

**Figure 2:**
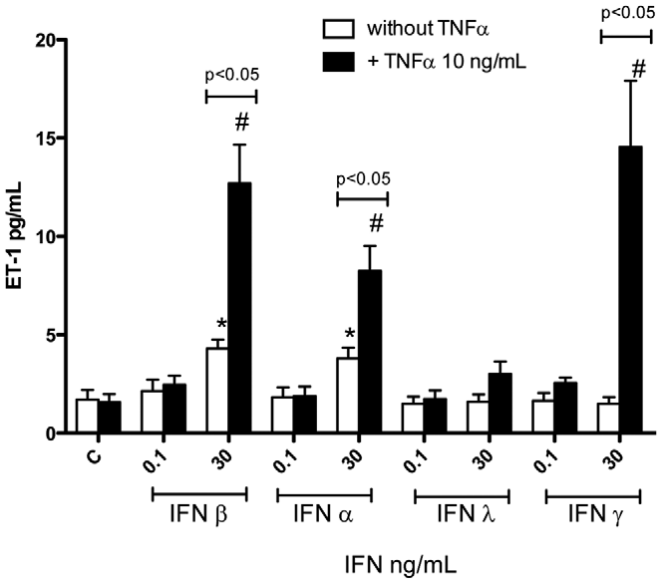
Effect of Type I–III IFNs on ET-1 release from HPASMCs. In the presence of TNFα, type I and type II, but not type III, interferons (IFNs) induce the release of ET-1 from human pulmonary artery smooth muscle cells (HPASMCs). Cells were treated with IFNs for 24 hours. Data are the mean ± standard error of the mean for n = 8. Within-group analysis was performed using one-way ANOVA followed by a Dunnett's post-test, where * indicates p<0.05 compared to control. Between-group analysis, for the effect of TNFα, was performed using two-way ANOVA followed by Bonferroni's post-test where # indicates p<0.05.

Under control culture conditions endothelial cells, including HLMVEC, release relatively high levels of ET-1. However, we have shown that other cell types, including vascular smooth muscle cells, release relatively low levels [22]–[26], [31]. We previously showed that vascular smooth muscle cells, including those from pulmonary arteries, release increased ET-1 when stimulated with the combination of IFNγ and TNFα [22]–[26], [31]. Here we have extended these observations and show that the type I IFNs, universal IFNα and IFNβ, induce ET-1 release without the need for TNFα, although this release is increased when TNFα is given as a pre-treatment (**Figure 2**). As we have found before, IFNγ, in the presence of TNFα, induced ET-1 release from HPASM cells (**Figure 2**). Interestingly, type III IFNλ had no effect on ET-1 release either in the absence or presence of TNFα (**Figure 2**). As we have found before, HLMVEC released relatively high levels of ET-1, which were not increased by treatment with any of the IFN preparations (data not shown).

### Effect of IFN preparations on IP10 and ET-1 release by human lung cells

**Figure 3:**
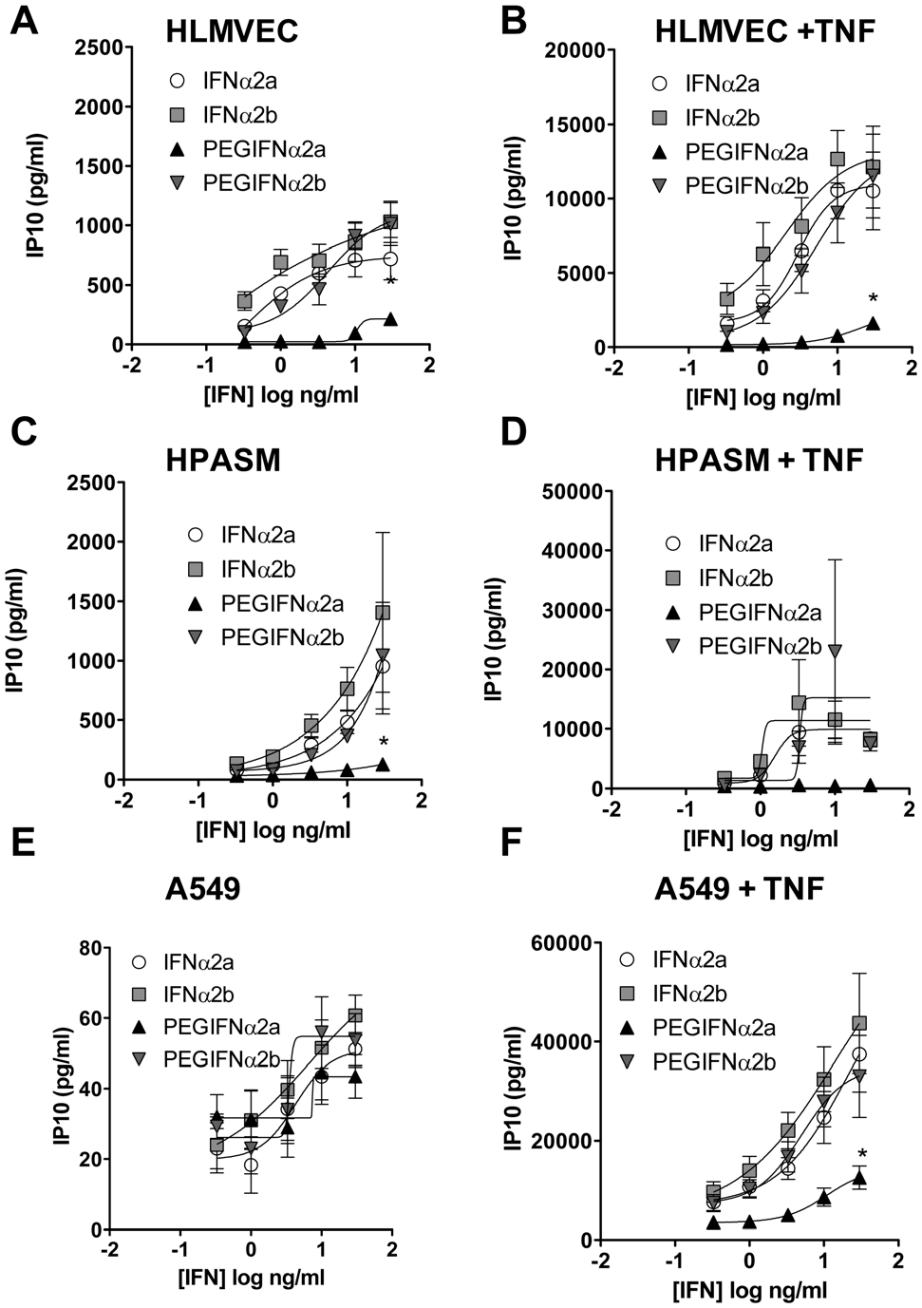
Effect of native versus pegylated IFN on IP10 release from HLMVECs. Effect of native IFNα2a or IFNα2b versus pegylated (PEG) forms on IP10 release from human lung microvascular endothelial cells (HLMVECs; A, B), human pulmonary artery smooth muscle cells (HPASMCs; C, D) and type II pneumocytes (A549 cells; E, F). Cells were treated with interferons (IFNs) alone (A, C and E) or in the presence of TNFα (10 ng/mL; B, D and F) for 24 hours. Data shown are mean ± standard error of the mean for n = 6 and were analysed using two-way ANOVA where * indicates p<0.05 for PEG-IFNα2a versus the other three IFNs.

As was seen with universal IFNα, authentic IFNα2a and IFNα2b activated HLMVEC and HPASM cells to release IP10 (**Figures 3A, C**). However, in A549 cells IFNα2a and IFNα2b were not able to induce high amounts of IP10 unless cells were pre-treated with TNFα (**Figure 3E**).

**Figure 4:**
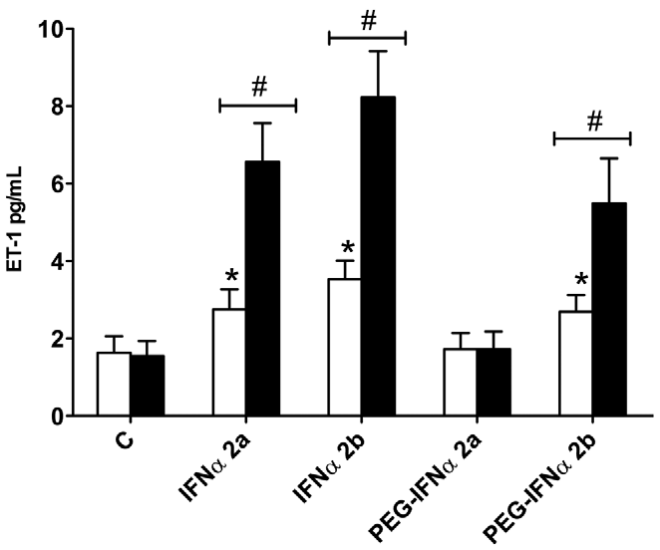
Effect of native versus pegylated IFN on ET-1 release from HPASMCs. = Effect of native 30 ng/mL IFNα2a or IFNα2b versus pegylated (PEG) forms on ET-1 release from human pulmonary artery smooth muscle cells (HPASMCs). Cells were treated with interferons (IFNs) for 24 hours. Data are the mean ± standard error of the mean for n = 8. Within-group analysis was performed using one-way ANOVA followed by a Dunnett's post-test, where * indicates p<0.05 compared to control. Between-group analysis, for the effect of TNFα was performed using two-way ANOVA followed by Bonferroni's post-test where # indicates p<0.05.

Similar to what was observed with non-pegylated IFNα2a and IFNα2b, 12KDa-PEGIFNα2b induced IP10 release from each cell type tested which, whilst robust, tended to be less potent than IFNα2b. However, in all models the induction of IP10 release with 40KDa-PEGIFNα2a was significantly less than with the other IFNα preparations tested. In line with observations made with IP10 release, ET-1 release from TNFα-primed HPASM cells was increased by IFNα2a, IFNα2b and 12KDa-PEGIFNα2b, but not by 40KDa-PEGIFNα2a (**Figure 4**).

In order to validate our observations using clinical preparations of pegylated IFNα, we performed a second set of experiments using different batches of 40KDa-PEGIFNα2a and 12KDa-PEGIFNα2b to observe the effect on IP10 release from HLMVEC and A549 cells treated with or without TNFα. Similar results were obtained to those shown in **Figure 3** (data not shown).

### Effect of IFN preparations on inflammatory gene expression

**Figure 5:**
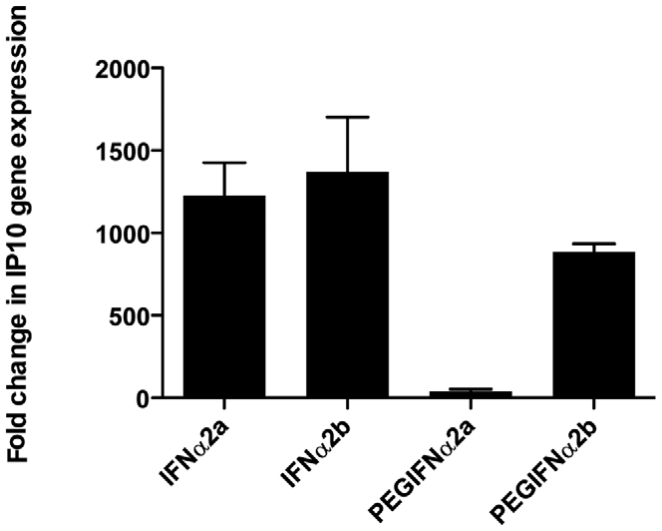
Effect of native versus pegylated IFN on IP10 gene induction in HLMVECs. Effect of native IFNα2a, IFNα2b versus pegylated (PEG) forms (40KDa-PEGIFNα2a and 12KDa-PEGIFNα2b) on IP10 gene induction in human lung microvascular endothelial cells (HLMVECs). Cells were treated with interferon (IFN) for 6 hours. Data are the mean ± standard error of the mean for n = 3.

Eighty-four inflammatory genes were measured in HLMVEC treated with IFNα2a, IFNα2b, 40KDa-PEGIFNα2a and 12KDa-PEGIFNα2b. A relatively small signature of genes were increased by the IFNα preparations (**Table S1**, available online); however, in line with our chemokine data, IP10 mRNA was strongly induced (**Figure 5**). Furthermore, in line with results for IP10 protein release, 40KDa-PEGIFNα2a was the weakest inducer of IP10 gene expression (**Figure 5**) when compared with authentic IFNα preparations or 12KDa-PEGIFNα2b (**Table S1**, available online).

### Differential effects of monopegylated isomers of 40KDa-PEGIFNα2a

**Figure 6:**
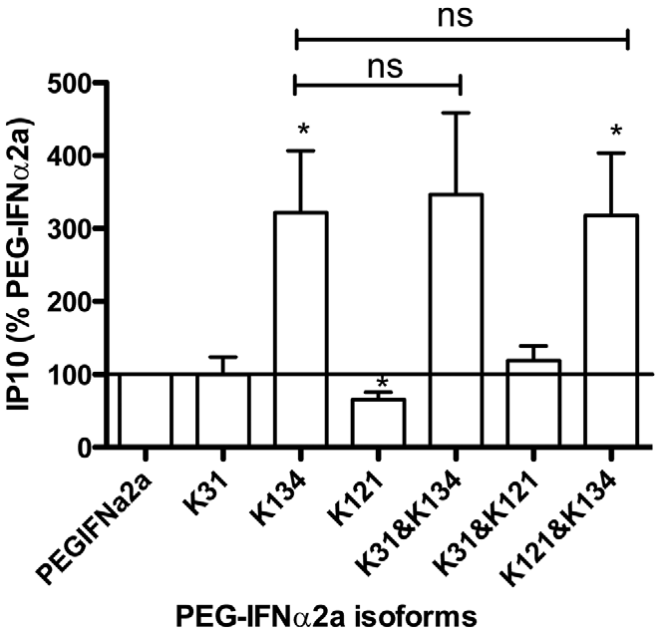
Effect of PEG-IFN α2a isoforms on IP10 release from HLMVECs. Effect of different isoforms of PEG-IFN α2a on IP10 release from human lung microvascular endothelial cells (HLMVECs) co-treated with TNFα (10 ng/mL). Cells were treated for 24 hours with PEGIFNα2a or isoforms of PEGIFNα2a (K31, K134, K121) each at 10 ng/mL. Data are mean ± standard error of the mean for n = 7. Data have been normalized using IP10 release in the presence of 40KDa-PEGIFNα2a as 100%, and were analysed using a paired one sample t-test for each isoform or combination compared to PEGIFNα2a, where * indicates p<0.05. Intergroup analysis was performed using one-way ANOVA followed by Bonferroni's Multiple Comparison Test, where # indicates p<0.05.

PEGIFNα formulations contain multiple monopegylated isomers with different antiviral activity and different affinities to IFNAR1 and IFNAR2 [5]. In order to better understand how 40KDa-PEGIFNα2a activates lung endothelial cells we investigated the efficacy of selected isoforms (K31, K34 and K121) on IP10 release by HLMVEC. These isoforms were previously found to have the following rank order of efficacy as antiviral agents *in vitro*: K31>K134>40KDa-PEGIFNα2a>K121 [5]. However, with regards to the global mRNA expression profile, a ranking of K134>K31>40KDa-PEGIFNα2a>K121 was established [5]. Similarly, in our study we found the same order (K134>K31 = 40KDa-PEGIFNα2a>K121) with regards to the propensity to induce IP10 release from human lung microvascular endothelial cells (10 ng/mL; **Figure 6**). Interestingly, the effect of the K134 isomer on IP10 release was not altered in the presence of K121 or K131, isomers with less IP10-inducing activity. This is in line with the different receptor affinities of the isomers postulated by Foser et al. [5] and suggests differential affinity for the K134 isomer by IFNAR1 and IFNAR2.

## Discussion

PEGIFNα plus ribavirin remains the cornerstone of treatment for all patients infected with the hepatitis C virus (HCV) despite the recent approval of two new direct-acting antivirals for the treatment of genotype 1 HCV [32], [33]. Side effects to PEGIFNα are common in CHC patients and include flu-like symptoms, myalgia, fatigue, gastrointestinal disturbances, psychiatric disorders and haematological abnormalities [34]–[36]. Interstitial pneumonitis is a rare adverse event observed in patients undergoing treatment for chronic HCV infection [6], [8], [9]. However, the case fatality rate is reported to be as high as 7%, and in the comprehensive review by Slavenburg, all fatal cases were attributed to 12KDa-PEGIFNα2b [6]. To date, no diagnostic biomarker has been identified for interstitial pneumonitis induced by type I IFNs, but several features appear to be shared among different forms of ILDs, including idiopathic pulmonary fibrosis (IPF), hypersensitivity pneumonitis and sarcoidosis. Specifically, ILDs are thought to be the result of the specific migration and the inflammatory effects of T-cells and macrophages, among others, to the lung. Migration of these cells to the lung is promoted by the secretion of chemokines in response to a stimulus in the lung. Among the immune cells found in murine models of ILDs, CXCR3+ T-cells in particular are found in inflamed lung tissue or murine models of interstitial lung disease. Consequently, studies have focussed on CXCR3 and its ligands IP10/CXCL10 and MIG/CXCL9. In an allograft model of lung injury, it was shown that blocking of IP10 and MIG reduced the inflammatory activity of pulmonary CXCR3+CD4+ Th1 cells on the lung, whereas their migration to the lung was not inhibited [11]. By contrast, in a murine model of *Pneumocystis pneumonia*, it was shown that overexpression of IP10 in lungs led to increased migration of CXCR3+CD8+ T-cells to the lung followed by accelerated *Pneumocystis* clearance [10]. Furthermore, it has also been shown that blocking of IP10 leads to the delayed clearance of lung infection with *Klebsiella pneumonia* [14].

Consistent with data from mouse studies, Nakayama and colleagues [16] found significantly elevated IP10 levels in BALF and elevated serum levels of epithelial-derived neutrophil-activating protein 78 (ENA-78; also termed CXCL5) in patients with nonspecific interstitial pneumonia compared to patients with IPF and controls. Whereas, patients with IPF showed higher ENA-78 levels in BALF compared to patients with nonspecific interstitial pneumonia and controls. In both cases correlations were found to the absolute number of lymphocytes in the BALF. Similarly, Katoh et al. [17] found elevated BALF levels of IP10 and MIG in patients with chronic eosinophilic pneumonitis. Furthermore, other investigators [15], [18] have found associations between IP10 and interstitial lung diseases. In addition to chemokines, several observations [18], [19] link ILD to angiogenic peptides such as ET-1. Therefore, IP10 and ET1 are presumably strong contributors to the pathophysiology of ILD and, conversely, could be helpful as safety biomarkers during IFNα therapy.

In the current study we found that universal IFNα, IFNα2a, IFNα2b, 40KDa-PEGIFNα2a and 12KDa-PEGIFNα2b, but not IFNλ, induced IP10 release and gene induction in human lung microvascular endothelial cells. IFNα2a, IFNα2b, 40KDa-PEGIFNα2a and 12KDa-PEGIFNα2b also induced IP10 release from human pulmonary artery smooth muscle cells and from TNFα-primed A549 cells. In each of these cell types 40KDa-PEGIFNα2a was less active than either native forms of IFNα2a and IFNα2b or 12KDa-PEGIFNα2b. Similarly IFNα2a, IFNα2b and 12KDa-PEGIFNα2b, but not 40KDa-PEGIFNα2a, induced ET-1 release from human pulmonary artery smooth muscle cells. Importantly, data with IP10 release were generated in a blinded fashion and confirmed by using different batches of 40KDa-PEGIFNα2a.

These observations are consistent with the PEG moiety causing steric hindrance and inhibiting the attachment of the IFN moiety to its receptor in vitro. The extent of this steric hindrance is related to the size and shape of the PEG moiety used: 12KDa-PEGIFNα2b retains approximately 30–40% of the specific activity of its unmodified parent IFNα2b [37], [38], whereas 40KDa-PEGIFNα2a retains approximately 0.5–7% of the specific activity of its unmodified parent IFNα2a [38], [39]. This was reflected in our assays in which 12KDa-PEGIFNα2b tended to be less potent than unmodified IFNα2b. However, spontaneous hydrolysis of 12KDa-PEGIFNα2b is known to occur [40] and this could also increase *in vitro* activity.

In the case of 40KDa-PEGIFNα2a, we found that compared to the parent compound approximately 20–100-fold more 40KDa-PEGIFNα2a was required to induce similar levels of IP10 release from cells. This is consistent with other reports that 40KDa-PEGIFNα2a is less potent than unmodified IFNα *in vitro* [4], [38]. However, precisely how much less active 40KDa-PEGIFNα2a is than unmodified IFNα in *in vitro* assays is dependent upon the assay used. Results from two different cell-based *in vitro* antiviral assays showed that 40KDa-PEGIFNα2a was either 14-fold or 200-fold weaker than unmodified IFNα [39]. Similarly, in a range of *in vitro* antiproliferative activity assays, 40KDa-PEGIFNα2a was found to be between 20- and 250-fold less potent than unmodified IFNα depending on the cell type used [39]. Similar findings were reported by Grace et al. who found that 40KDa-PEGIFNα2a was approximately 20- to 30-fold less potent than 12KDa-PEGIFNα2b in FS-71 or A549 antiviral protection assays, respectively, or approximately 40-fold less potent in an antiproliferation assay [37].

The reason why the potency of 40KDa-PEGIFNα2a compared to unmodified IFNα or 12KDa-PEGIFNα2b varies in *in vitro* assays is not clear but may be related to the biology of individual isoforms present in clinical preparations [5]. Importantly, the 40KDa-PEGIFNα2a used in the present study is a mixture of at least nine monopegylated isomers of IFNα2a that have a range of differing antiviral, antiproliferative and gene-induction-specific activities [5]. For example, the 40KDa-PEGIFNα2a isoform K31 has higher antiviral activity yet lower gene induction activity than the isoform K134 [5]. In line with this we also found that K134 induced higher IP10 levels than K31 from human lung endothelial cells. Hence the isomer composition of therapeutic IFNα preparations may impact on efficacy and/or safety. This has direct and important relevance for the safety assessment in the increasing market of biosimilar IFNα preparations. Here it has to be acknowledged that biosimilars, which are produced using different manufacturing processes, may not have the same profile of isomers and hence may have clinically relevant differences in safety and/or efficacy profiles. However, the use of simple human tissue screens, such as lung endothelium, in parallel with classical *in vitro* antiviral efficacy assays, may help to identify the optimal balance of isomers of PEGIFNα with potent antiviral effects, but reduced deteriorating actions on stromal tissues.

Interestingly, effects of IFNα preparations on IP10 release were greatly increased when cells were pre-treated with TNFα. Indeed, IP10 release by A549 cells was negligible unless the cells were first primed with TNFα. This observation is somewhat in keeping with data from Sanda and colleagues who found negligible induction of the IP10 gene after IFNα (1.16-fold) or IFNγ (2.53-fold) treatment alone [41], whereas Clarke et al. demonstrate that TNFα synergizes with IFNγ to induce IP10 in human airway smooth muscle [42]. In airway smooth muscle cells the synergistic effects of TNFα and IFNγ were mediated via STAT-1, NF-κB and the transcriptional coactivator CREB-binding protein [42]; the role of these pathways in synergies of TNFα with type I IFNα and IFNβ for IP10 induction remains the subject of investigation. However, our findings are also in line with a recent report showing that TNF activates a positive feedback loop via IFNβ and interferon regulatory factor 1 (IRF-1), which in turn leads to up-regulation of chemokines, including IP10 [43].

With regards to ET-1, Wort et al. have previously shown that HPASM cells, which normally release very low levels of ET-1, release increased levels after treatment with TNFα and type II IFNγ [22]–[25]. In the present study we extend these findings, bringing clinical relevance to the observations by showing that, in addition to type II IFNγ, type I IFNα and IFNβ induce ET-1 release from HPASM cells primed with TNFα. Interestingly, and in line with the IP10 data, 40KDa-PEGIFNα2a showed no propensity to induce ET-1, whereas elevated levels were induced in HPASM cells by both non-pegylated IFNα and 12KDa-PEGIFNα2b. Again, these differences are likely due to steric hindrance of the 40KDa-PEG moiety restricting access of IFN to its receptor. Furthermore, we found that, in contrast to observations with IFNγ, for ET-1 release induced by type I IFNs, there was no requirement for TNFα. The signalling pathways leading to ET-1 release from vascular smooth muscle cells have been elucidated for type II IFNγ, where enhanced NF-κB [26] binding and histone acetylation at specific κB sites are thought to be involved [24]. The role of these pathways in ET-1 release by type I IFNs remains to be investigated.

IP10 is classically associated with type II IFNγ and, as such, was first described as ‘IFNγ inducible protein 10’ [44]. While several reports [43], [45], [46] indicate systemic induction of IP10 by IFNα in mice and in human peripheral blood mononuclear cells, to our knowledge there are only a limited number of studies reporting IP10 induction after type I IFNα or IFNβ treatment in human endothelial cells [21], [47]. In their study, Indraccolo and colleagues showed similar results to the current study and found that IFNα, IFNβ and IFNγ induced IP10 release and gene induction in human umbilical vein and dermal microvascular endothelial cells [21]. To our knowledge, our study is the first to show this phenomenon in human lung endothelial cells or in pulmonary vascular smooth muscle cells. In addition to IP10 we also found that IFNα induced clear increases in CXCL11 gene expression. In our study validation of gene induction with CXCL11 protein release was not performed due to lack of material. These experiments would strengthen our work; however, our findings are in keeping with those of Indraccolo et al. who showed CXCL11 induction and protein release after type I and type II IFN treatment of human umbilical vein endothelial cells [21].

The lack of effect of IFNλ on IP10 and ET-1 release by human lung cells is interesting and potentially clinically relevant. PEGIFNλ is currently undergoing clinical trials for the treatment of chronic hepatitis C and early clinical indications suggest that it is efficacious [48]. IFNλ activates IFNλ receptor 1 and interleukin-10 receptor 2, which are far more restricted in their tissue distribution than the IFNAR or IFNγ receptor complexes [48]. Our data suggest that receptors for IFNα, IFNβ and IFNγ are present on the HPASM and HLMVEC cells used in the present study, but that receptors for IFNλ are not. The relationship between IFN receptor expression and activation of inflammatory responses in human lung cells remains the subject of investigation.

In HCV-infected patients, pre-treatment levels of IP10 have been shown to be inversely correlated with sustained virological response rates [49]. Our data are consistent with the notion that IP10 levels could be an indicator of the level and activity of intrinsic type I IFN pathways activated by viral infection. Conversely, a high pre-treatment IFN activation status, as possibly indicated by high IP10 values, could in part render the host non-responsive towards exogenous IFNα therapy. Therefore, in the context of interstitial pneumonitis, an interesting question remains whether high pre-treatment levels of IP10 (and/or ET-1) levels could predispose patients to an altered risk of IFN-induced interstitial pneumonitis under IFNα therapy. In addition, the findings of a “sensitizing” effect of TNF-α on the IFN-responsiveness and IP10 induction in HLMVEC, HPASM and A549 cells pose the interesting question of whether underlying inflammatory conditions in the lung could predispose IFN-treated patients to pneumonitis or other ILDs. Our data are consistent with the idea that host mediators, including IP10 and ET-1, could be useful biomarkers for lung toxicity induced by IFN therapy. However, this work uses simple cell-based in vitro models, which, although useful, are unlikely to mimic the complexity of the in vivo situation. This is also the case in our study where, as discussed above, PEG is known to cause steric hindrance of the IFN to its receptors in in vitro assays, but that this phenomenon is independent of its pharmacology in vivo where the PEG serves to stabilize the IFN. Furthermore, there is the added complication, as discussed above, that HCV will lead to increased IP10 (and potentially ET-1) levels due to host IFN release, thus impeding the use of IP10 as biomarker for interstitial pneumonitis. Indeed, recent pilot work from our group, where ET-1 levels were measured in the plasma of archived samples of 16 patients during treatment with PEGIFNα2, showed increases in samples of 2 patients [50]. However, in this study, lung function was not measured. Thus, we suggest that before this idea can be fully appreciated clinical studies should be performed where biomarkers, including IP10 and ET-1, are measured in patients receiving IFN therapy and correlated with lung function over time.

## Supporting Information

Table S1 **Effect of IFNα2a, IFNα2b, 40KDa-PEGIFNα2a and 12KDa-PEGIFNα2b on gene induction; n = 3.**
(DOCX)
