# Supplementary material for: Effect of Different Interferonα2 Preparations on IP10 and ET-1 Release from Human Lung Cells
Source: PLoS One. 2012 Oct 8;7(10):e46779. doi: 10.1371/journal.pone.0046779 (PMC3466308; doi:10.1371/journal.pone.0046779)
Supplement: Table S1 — Effect of IFNα2a, IFNα2b, 40KDa-PEGIFNα2a and 12KDa-PEGIFNα2b on gene induction; n = 3. (DOCX) [file pone.0046779.s001.docx]

**Table S1: Effect of IFNα2a, IFNα2b, 40KDa-PEGIFNα2a and 12KDa-PEGIFNα2b on gene induction; n = 3.**

| **IFNα2a** | | **IFNα2b** | | **PEGIFNα2a** | | **PEGIFNα2b** | |
| --- | --- | --- | --- | --- | --- | --- | --- |
|  | **Mean (sem)** |  | **Mean (sem)** |  | **Mean (sem)** |  | **Mean (sem)** |
| CXCL10 | 1226.107 (199.562) | CXCL10 | 1369.629 (332.274) | CXCL10 | 39.576 (12.914) | CCL18 | 1917.754 (1917.426) |
| CXCL11 | 493.878 (163.920) | CXCL11 | 612.368 (220.978) | CCL5 | 36.413 (33.788) | CXCL10 | 884.015 (50.507) |
| CXCL9 | 152.226 (87.117) | CXCL9 | 150.503 (61.556) | IL1F6 | 35.250 (34.360) | CXCL11 | 340.53 (51.954) |
| CCL8 | 102.572 (40.978) | CCL8 | 115.373 (66.827) | CEBPB | 33.537 (31.081) | CXCL9 | 125.054 (68.164) |
| CCL5 | 58.822 (53.764) | CCL5 | 66.238 (62.556) | CXCL11 | 23.070 (10.016) | CCL8 | 57.636 (21.598) |
| CCL7 | 28.366 (17.185) | CCL7 | 28.040 (19.340) | CXCL5 | 18.261 (15.995) | CCL5 | 55.544 (50.503) |
| CXCL5 | 15.367 (13.409) | CXCL5 | 21.763 (20.031) | CXCL1 | 6.730 (3.422) | CCL7 | 23.944 (13.752) |
| CEBPB | 14.522 (12.304) | CEBPB | 15.004 (13.331) | CCL7 | 5.290 (2.785) | CXCL5 | 20.656 (18.719) |
| CCL17 | 7.702 (6.863) | CXCL1 | 5.423 (3.012) | C3 | 3.192 (1.539) | CEBPB | 14.747 (12.094) |
| CXCL1 | 5.232 (2.435) | LTA | 4.017 (3.204) | MIF | 3.041 (1.149) | XCR1 | 5.954 (4.673) |
| C3 | 4.110 (1.774) | C3 | 3.916 (2.562) | CCL23 | 2.989 (1.220) | CXCL1 | 4.656 (2.150) |
| IL5 | 3.929 (2.657) | CCL24 | 3.212 (1.459) | CXCL12 | 2.950 (1.820) | MIF | 3.099 (1.182) |
| CCL23 | 3.840 (1.361) | CCL23 | 2.646 (0.808) | CCL8 | 2.939 (1.546) | IL5 | 2.517 (1.081) |
| MIF | 3.087 (1.257) | MIF | 2.639 (1.191) | IL17C | 2.706 (0.961) | IL17C | 2.442 (1.198) |
| CXCL3 | 2.989 (1.487) | IL1R1 | 2.259 (0.662) | CXCL3 | 2.310 (0.924) | CCL24 | 2.411 (1.295) |
| IL17C | 2.573 (0.905) | CXCL3 | 2.234 (0.886) | IL10RB | 2.262 (0.490) | CCL23 | 2.359 (0.508) |
| IL1R1 | 2.407 (1.017) | IL10RB | 2.142 (0.422) | IL1R1 | 2.251 (0.989) | IL1R1 | 2.342 (0.802) |
| IL10RB | 2.238 (0.233) | CXCL14 | 2.103 (1.312) | BCL6 | 2.208 (1.058) | CXCL12 | 2.321 (1.390) |
| LTB4R | 2.086 (0.765) | BCL6 | 1.935 (0.861) | IL13RA1 | 1.950 (0.883) | LTB4R | 2.167 (0.855) |
| CCL16 | 2.057 (0.692) | XCR1 | 1.922 (1.405) | C4A | 1.921 (0.343) | BCL6 | 1.959 (0.798) |
| CCL2 | 1.934 (0.356) | CXCL12 | 1.902 (0.902) | TOLLIP | 1.919 (0.492) | C3 | 1.935 (0.784) |
| CXCL12 | 1.902 (1.135) | CCL2 | 1.837 (0.621) | CXCL6 | 1.821 (0.737) | CXCL3 | 1.848 (0.508) |
| CCL24 | 1.872 (0.719) | IL13RA1 | 1.833 (0.967) | LTB4R | 1.708 (0.183) | SPP1 | 1.807 (0.455) |
| IL13RA1 | 1.783 (0.606) | LTB4R | 1.650 (0.176) | ABCF1 | 1.680 (0.530) | IL10RB | 1.799 (0.368) |
| BCL6 | 1.721 (0.439) | IL1RN | 1.578 (0.582) | IL1A | 1.631 (0.331) | IL13RA1 | 1.798 (0.692) |
| LTA | 1.702 (1.170) | ABCF1 | 1.539 (0.489) | LTB | 1.624 (0.518) | C5 | 1.727 (0.590) |
| CARD18 | 1.683 (1.226) | LTB | 1.518 (0.522) | IL1B | 1.603 (0.433) | LTB | 1.616 (0.540) |
| LTB | 1.637 (0.577) | CXCL2 | 1.483 (0.417) | CCL24 | 1.595 (0.751) | C4A | 1.609 (0.344) |
| IL1B | 1.628 (0.212) | CCL11 | 1.463 (0.689) | C5 | 1.542 (0.281) | IL1A | 1.572 (0.061) |
| XCR1 | 1.600 (1.273) | CXCL6 | 1.388 (0.538) | CCL20 | 1.542 (0.595) | LTA | 1.517 (0.564) |
| ABCF1 | 1.565 (0.319) | IL1B | 1.374 (0.221) | LTA | 1.423 (0.728) | IL1RN | 1.486 (0.361) |
| C4A | 1.557 (0.560) | IL1A | 1.288 (0.348) | IL8 | 1.407 (0.210) | CCL2 | 1.480 (0.226) |
| CCR4 | 1.533 (0.863) | CCL13 | 1.234 (0.478) | CCL2 | 1.384 (0.369) | CXCL2 | 1.435 (0.305) |
| SPP1 | 1.465 (0.171) | CCL17 | 1.221 (0.400) | CCL21 | 1.291 (0.409) | TOLLIP | 1.354 (0.369) |
| IL1F6 | 1.393 (0.779) | IL10 | 1.190 (0.390) | SCYE1 | 1.226 (0.406) | CXCL6 | 1.283 (0.556) |
| CXCL6 | 1.383 (0.482) | IL17C | 1.179 (0.461) | CXCL2 | 1.225 (0.364) | IL1B | 1.262 (0.128) |
| CXCL2 | 1.364 (0.391) | C4A | 1.165 (0.081) | SPP1 | 1.223 (0.267) | ABCF1 | 1.253 (0.277) |
| CCL21 | 1.349 (0.508) | SCYE1 | 1.112 (0.248) | IL5 | 1.015 (0.528) | SCYE1 | 1.207 (0.459) |
| IL1A | 1.345 (0.079) | SPP1 | 1.107 (0.326) | XCR1 | 0.951 (0.681) | CCL16 | 1.108 (0.500) |
| IL1RN | 1.336 (0.485) | CCL1 | 1.097 (0.058) | CCL1 | 0.905 (0.418) | CCL21 | 1.105 (0.155) |
| TOLLIP | 1.299 (0.208) | CCL21 | 1.096 (0.065) | CARD18 | 0.863 (0.254) | TNF | 1.081 (0.296) |
| IL10 | 1.293 (0.701) | CCR4 | 1.061 (0.349) | IL10 | 0.838 (0.258) | CCR4 | 1.080 (0.157) |
| C5 | 1.209 (0.600) | TOLLIP | 1.003 (0.312) | CCL11 | 0.833 (0.259) | CCL1 | 1.022 (0.421) |
| SCYE1 | 1.170 (0.324) | C5 | 0.942 (0.107) | IL10RA | 0.786 (0.274) | CCL15 | 0.984 (0.663) |
| CCR7 | 1.144 (0.696) | IL1F5 | 0.889 (0.234) | CCR7 | 0.774 (0.175) | CCL11 | 0.928 (0.469) |
| IL8 | 1.098 (0.157) | CCL20 | 0.867(0.296) | CCL16 | 0.768 (0.281) | IL8 | 0.899 (0.497) |
| IL1F10 | 1.035 (0.143) | CARD18 | 0.864 (0.226) | CCL13 | 0.768 (0.281) | IL5RA | 0.864 (0.506) |
| CCL1 | 0.985 (0.118) | IL8 | 0.862 (0.102) | CCL15 | 0.768 (0.281) | IL1F5 | 0.841 (0.521) |
| IL1F9 | 0.983 (0.165) | IL1F9 | 0.837 (0.250) | CCL18 | 0.768 (0.281) | CCL13 | 0.840 (0.521) |
| IL1F8 | 0.955 (0.112) | IL10RA | 0.825 (0.240) | CCL19 | 0.768 (0.281) | CCL19 | 0.840 (0.521) |
| CCL13 | 0.868 (0.137) | CCR7 | 0.818 (0.068) | CCL4 | 0.768 (0.281) | CCL4 | 0.840 (0.521) |
| IL1F5 | 0.865 (0.578) | CCL15 | 0.803 (0.219) | CCR2 | 0.768 (0.281) | CCR2 | 0.840 (0.521) |
| CCL20 | 0.848 (0.230) | CCL18 | 0.803 (0.219) | CCR5 | 0.768 (0.281) | CCR5 | 0.840 (0.521) |
| IL1F7 | 0.799 (0.054) | CCL19 | 0.803 (0.219) | CCR6 | 0.768 (0.281) | CCR6 | 0.840 (0.521) |
| IL10RA | 0.765 (0.295) | CCL4 | 0.803 (0.219) | CCR8 | 0.768 (0.281) | CCR8 | 0.840 (0.521) |
| CCR9 | 0.703 (0.287) | CCR2 | 0.803 (0.219) | CCR9 | 0.768 (0.281) | CCR9 | 0.840 (0.521) |
| CCL18 | 0.692 (0.278) | CCR5 | 0.803 (0.219) | CRP | 0.768 (0.281) | CRP | 0.840 (0.521) |
| CCL11 | 0.692 (0.278) | CCR6 | 0.803 (0.219) | CX3CR1 | 0.768 (0.281) | CX3CR1 | 0.840 (0.521) |
| CCL15 | 0.692 (0.278) | CCR8 | 0.803 (0.219) | CXCL13 | 0.768 (0.281) | CXCL13 | 0.840 (0.521) |
| CCL19 | 0.692 (0.278) | CCR9 | 0.803 (0.219) | CXCL14 | 0.768 (0.281) | CXCL14 | 0.840 (0.521) |
| CCL4 | 0.692 (0.278) | CRP | 0.803 (0.219) | CXCL9 | 0.768 (0.281) | CARD18 | 0.840 (0.521) |
| CCR2 | 0.692 (0.278) | CX3CR1 | 0.803 (0.219) | IL13 | 0.768 (0.281) | IL10RA | 0.840 (0.521) |
| CCR5 | 0.692 (0.278) | CXCL13 | 0.803 (0.219) | IL1F10 | 0.768 (0.281) | IL13 | 0.840 (0.521) |
| CCR6 | 0.692 (0.278) | IL13 | 0.803 (0.219) | IL1F8 | 0.768 (0.281) | IL1F10 | 0.840 (0.521) |
| CCR8 | 0.692 (0.278) | IL1F10 | 0.803 (0.219) | IL1F9 | 0.768 (0.281) | IL1F6 | 0.840 (0.521) |
| CRP | 0.692 (0.278) | IL1F6 | 0.803 (0.219) | IL1RN | 0.768 (0.281) | IL1F8 | 0.840 (0.521) |
| CX3CR1 | 0.692 (0.278) | IL1F8 | 0.803 (0.219) | IL22 | 0.768 (0.281) | IL1F9 | 0.840 (0.521) |
| CXCL13 | 0.692 (0.278) | IL22 | 0.803 (0.219) | IL5RA | 0.768 (0.281) | IL22 | 0.840 (0.521) |
| CXCL14 | 0.692 (0.278) | IL5 | 0.803 (0.219) | IL8RA | 0.768 (0.281) | IL8RA | 0.840 (0.521) |
| IL13 | 0.692 (0.278) | IL5RA | 0.803 (0.219) | IL8RB | 0.768 (0.281) | IL8RB | 0.840 (0.521) |
| IL22 | 0.692 (0.278) | IL8RA | 0.803 (0.219) | IL9 | 0.768 (0.281) | IL9 | 0.840 (0.521) |
| IL5RA | 0.692 (0.278) | IL8RB | 0.803 (0.219) | CD40LG | 0.768 (0.281) | CD40LG | 0.840 (0.521) |
| IL8RA | 0.692 (0.278) | IL9 | 0.803 (0.219) | IFNA2 | 0.688 (0.324) | IL10 | 0.836 (0.519) |
| IL8RB | 0.692 (0.278) | CD40LG | 0.803 (0.219) | IL1F5 | 0.686 (0.325) | IFNA2 | 0.765 (0.551) |
| IL9 | 0.692 (0.278) | CCL16 | 0.778 (0.222) | TNF | 0.681 (0.146) | CCL17 | 0.726 (0.402) |
| CD40LG | 0.692 (0.278) | IL1F7 | 0.714 (0.148) | CCL17 | 0.630 (0.143) | CCR7 | 0.713 (0.397) |
| CCL26 | 0.621 (0.230) | TNF | 0.691 (0.132) | CCR4 | 0.579 (0.128) | CCL20 | 0.690 (0.134) |
| TNF | 0.584 (0.202) | IFNA2 | 0.682 (0.254) | CCL26 | 0.575 (0.121) | CCL26 | 0.631 (0.249) |
| IFNA2 | 0.577 (0.289) | CCL25 | 0.676 (0.253) | IL9R | 0.558 (0.162) | IL9R | 0.625 (0.316) |
| IL9R | 0.513 (0.207) | CCL26 | 0.655 (0.063) | CCR3 | 0.406 (0.068) | IL1F7 | 0.613 (0.257) |
| CCL3 | 0.461 (0.230) | IL9R | 0.578 (0.106) | IL1F7 | 0.284 (0.056) | CCR3 | 0.371 (0.163) |
| CCR3 | 0.363 (0.091) | CCL3 | 0.563 (0.287) | CCL25 | 0.196 (0.086) | CCL25 | 0.292 (0.144) |
| CCL25 | 0.358 (0.182) | CCR3 | 0.523 (0.087) | CCR1 | 0.095 (0.015) | CCL3 | 0.249 (0.135) |
| CCR1 | 0.272 (0.182) | CCR1 | 0.165 (0.043) | CCL3 | 0.075 (0.051) | CCR1 | 0.178 (0.080) |
